# Evaluation of proliferation and apoptosis markers in circulating tumor cells of women with early breast cancer who are candidates for tumor dormancy

**DOI:** 10.1186/s13058-014-0485-8

**Published:** 2014-11-29

**Authors:** Maria Spiliotaki, Dimitris Mavroudis, Kyriaki Kapranou, Harris Markomanolaki, Galatea Kallergi, Filippos Koinis, Kostas Kalbakis, Vassilis Georgoulias, Sofia Agelaki

**Affiliations:** 1 Laboratory of Tumor Cell Biology, School of Medicine, University of Crete, Voutes University Campus, Heraklion, 71003 Crete Greece; 2 Department of Medical Oncology, University General Hospital of Heraklion, Voutes, Heraklion, 71110 Crete Greece

## Abstract

**Introduction:**

Clinical dormancy is frequently observed in breast cancer. In the present study, we aimed to characterize circulating tumor cells (CTCs) in dormancy candidates (DC) with early breast cancer in terms of proliferation and apoptosis.

**Methods:**

Cytospins of peripheral blood mononuclear cells (PBMCs) were obtained from DC (n = 122) who were disease-free for at least 5 years and from metastatic patients (n = 40) who relapsed more than 5 years after surgery. Sequential samples from eight DC (n = 36) who maintained a prolonged disease-free status and from eight DC (n = 27) presenting late relapse during follow-up, were also analyzed. PBMCs were triple stained with a pancytokeratin, antibody along with anti-Ki67 and anti-M30 antibodies as proliferation and apoptosis markers, respectively.

**Results:**

CTCs were identified in 40 (33%) of 122 DC and in 15 (37.5%) of 40 metastatic patients. In total, twenty-five (62.5%) DC had exclusively dormant (Ki67(-)/M30(-)), seven (17.5%) had proliferative Ki67(+)/M30(-), four (10%) had apoptotic Ki67(-)/M30(+) and four (10%) had both phenotypes of proliferative and apoptotic CTCs. In comparison, 53.4% of CTC-positive metastatic patients had exclusively dormant and 46.6% had proliferative CTCs; none had apoptotic CTCs (*P* = 0.039). Among all CTCs detected in DC patients, 82.4% were dormant, whereas in the nondormant population, 32.5% were proliferative and 67.5% apoptotic. The respective percentages in metastatic patients were 59.1%, 100% and 0% (*P* <0.0001). Moreover, apoptotic CTCs prevailed among nondormant CTCs detected in sequential samples from DC who remained in a prolonged disease-free status compared to those presenting late relapse during follow-up (70.6% versus 43.5% (*P* = 0.0002)).

**Conclusions:**

The apoptotic index of CTCs is increased during clinical dormancy, whereas the proliferation index is increased on relapse. In addition, apoptotic CTCs are more frequently encountered during follow-up in DC patients who remain disease-free compared to those with subsequent late relapse, suggesting that monitoring proliferation and apoptosis in CTCs during clinical dormancy merits further investigation as a tool for predicting late disease recurrence.

**Electronic supplementary material:**

The online version of this article (doi:10.1186/s13058-014-0485-8) contains supplementary material, which is available to authorized users.

## Introduction

In breast cancer, relapses frequently occur many years or even decades after surgical removal of the primary tumor [1]–[3]. During the interval preceding relapse, patients have no clinical or radiological evidence of metastases by the use of standard work-up evaluations. In these patients, recurrence is thought to originate from cells that disseminated from the primary tumor and underwent a period of disease inactivity, termed dormancy, followed by a second period of active growth. These disseminated tumor cells can persist either as solitary dormant cells, which are quiescent, undergoing neither cell division nor apoptosis [4]–[6], or as dormant micrometastases, in which proliferation is balanced by apoptosis [7]. This hypothesis has been supported by mathematical modeling of breast cancer recurrence, showing that dormancy represents a period of cancer quiescence followed by active growth, rather than a phase of linear tumor progression [8]–[10].

The detection of circulating tumor cells (CTCs) in the blood or disseminated tumor cells (DTCs) in the bone marrow in clinically disease-free patients with early breast cancer as well as their association with worse patient prognosis has been well established [11]–[16]. Furthermore, after the completion of adjuvant treatment, a significant percentage of patients still harbor detectable tumor cells in the blood and/or bone marrow, and their presence remains an unfavorable prognostic factor [17]–[23]. In these studies, patients were evaluated shortly after the completion of adjuvant treatment or within 3 years after primary diagnosis [17],[18],[21] when the risk of recurrence is presumably higher [24]. However, in breast cancer, especially for patients with hormone receptor-positive disease undergoing adjuvant hormone therapy for 5 or more years, more than one-half of all recurrences and deaths occur beyond 5 years from diagnosis [3],[25] whereas, from 10 to 20 years, the rate of relapse is relatively steady at about 1.5% yearly [26],[27].

In a report by Meng *et al*., CTCs were detected in breast cancer patients who had no evidence of disease, 7 to 22 years after mastectomy [28]. In a recent study that included 312 patients with early breast cancer monitored for cytokeratin (CK)-19 mRNA positivity in peripheral blood during follow-up, we reported that 53.8% of patients had detectable CK-19 mRNA CTCs on at least one time point between the third and fifth year of follow-up and that persistence of CK-19 mRNA-positive CTCs during the first 5 years was associated with increased risk for late relapse and death [29]. However, it is evident that despite the presence of CTCs the development of metastases is not universal in all patients; the early recognition of patients who are at increased risk for recurrence remains an unmet need.

Although the precise mechanisms of breast cancer dormancy are still unclear, cellular markers are available to identify dormant CTCs. In experimental models, dormant cells are described as viable cells lacking the expression of both proliferative and apoptotic markers [5],[30]. In addition, evidence exists for a link between tumor dormancy and apoptosis [7], whereas in another report, a dormant tumor population was generated by balanced cell replication and cell death [28]. In the current study, we sought to detect CTCs in dormancy candidate (DC) patients with breast cancer, which were defined as patients who remained free of disease for at least 5 years following surgery, and to characterize their apoptotic and proliferative status by the use of a triple immunofluorescence method. Our results demonstrate that monitoring proliferation and apoptosis in CTCs could serve as a useful tool for the long-term follow-up of primary breast cancer patients.

## Methods

### Patients

Women with stage I to III early breast cancer (n = 122) who were under surveillance and had not experienced disease relapse during the first 5 years of follow-up (defined as dormancy candidates; DC), were eligible for this study. All patients had received adjuvant chemotherapy mostly in the context of research protocols of the Hellenic Oncology Research Group. After completion of adjuvant chemotherapy, patients received adjuvant radiotherapy and hormonal therapy when indicated according to their individual disease characteristics. There were no subgroups of patients who received hormone therapy only or no systemic therapy at all.

Patients’ follow-up consisted of pertinent medical history and physical examination, with laboratory and imaging studies restricted as indicated, every 3 months for the first 2 years, every 6 months for the next 3 years and yearly thereafter. Breast cancer patients (n = 40) presenting metastatic relapse more than 5 years after surgery were evaluated before the initiation of any systemic first-line therapy as a control group. In addition, sequential follow-up samples were evaluated in 16 out of 40 CTC DC identified as CTC-positive; eight of them had experienced late disease relapse and eight had remained disease-free during the whole follow-up period. Peripheral blood was also drawn from healthy female donors (n = 15) who had neither known illness at the time of sampling nor any history of malignant disease to ensure the specificity of the methods used. All patients and healthy volunteers gave their informed consent to participate in the study, which has been approved by the Ethics and Scientific Committees of the University General Hospital of Heraklion.

### Cell cultures

The breast cancer cell lines SKBR3 and MDA-MB231 were obtained from the American Type Culture Collection (Manassas, VA, USA). Cells were centrifuged on cytospins according to the procedure followed for patients’ samples to be used as controls for CK, M30 and Ki67 staining experiments, respectively.

SKBR3 cells were cultured in McCoy’s 5A GlutaMAX supplemented with 10% fetal bovine serum (FBS) (Gibco BRL Life Technologies, Rockville, MD, USA). MDA-MB231 cells were cultured in Dulbecco’s modified Eagle’s medium (DMEM) GlutaMAX supplemented with 10% FBS. Cells were maintained in a humidified atmosphere of 5% CO_2_ in air. Subcultivation was performed with 0.25% trypsin and 5 mM EDTA (Gibco BRL Life Technologies).

SKBR3 cells were cultured in the presence or absence of staurosporine 2μΜ (Merck, Darmstadt, Germany) for 2 hours to induce apoptotic events in drug-treated cells [31]. Twenty to twenty-four hours prior to the experiments, cells were transferred in serum-free medium. After incubation with staurosporine, cells were centrifuged on cytospins according to the same procedure followed for patients’ samples and were used as positive controls for CK and M30 expression.

Cyto-centrifuged MDA-MB231 cells were used as positive controls for CK and Ki67 expression. All experiments were performed during the logarithmic growth phase of cells.

### Sample collection and cytospin preparation

Twenty milliliters of blood were obtained from each patient and healthy volunteers. To avoid blood contamination by epithelial cells from the skin, all blood samples were collected after the first 5 ml of blood were discarded. Peripheral blood mononuclear cells (PBMCs) were isolated with Ficoll-Hypaque density gradient (d =1.077 g/mol) centrifugation at 1,800 rpm for 30 minutes. PBMCs were washed three times with phosphate-buffered saline (PBS) solution and centrifuged at 1,500 rpm for 10 minutes. Aliquots of 500,000 cells were centrifuged at 2.000 rpm for 2 minutes on glass slides. Cytospins were dried up and stored at -80°C. A total of 10^6^ PBMCs were analyzed per patient. Results are expressed as number of CTCs/500,000 PBMCs.

### Characterization of M30 and Ki67 staining on SKBR3 and MDA-MB231 breast cancer cell lines

**Figure 1:**
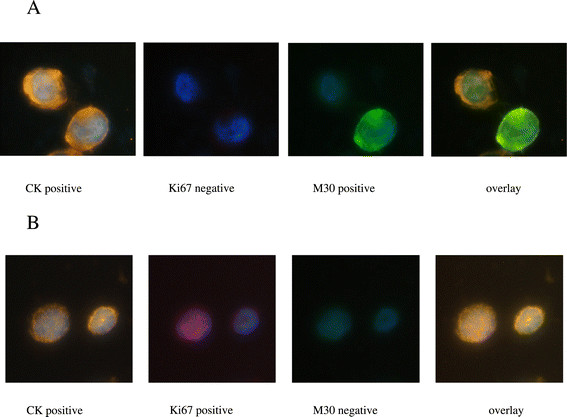
**Expression of cytokeratin and M30 or cytokeratin and Ki67 in SKBR3 and MDA-MB231 breast cancer cell lines respectively. (A)** Staurosporine-treated SKBR3 cells were triple stained with pancytokeratin rabbit antibody/secondary anti-rabbit Alexa Fluor 555 (orange), anti-Ki67 mouse antibody/secondary anti-mouse Alexa 633 (red) and M30 mouse FITC-conjugated antibody (green). Cell nuclei were stained with DAPI (blue). Images were obtained by the use of ARIOL system (X60). **(B)** MDA-MB231 cells were triple stained as described above. The positive nuclear dotted staining (red) was evaluated for Ki67 staining. Images were obtained by the use of ARIOL system (X60). ARIOL system, automated image analysis system; DAPI, 4’,6-diamidino-2-phenylindole; FITC, fluorescein isothiocyanate.

To evaluate apoptosis with a staining procedure in a model system, SKBR3 cells treated in the presence or absence of staurosporine were used. Apoptosis was determined by staining with the M30 fluorescein-conjugated mouse monoclonal antibody (CytoDEATH fluorescein; Roche, Manheim, Germany). M30 recognizes the respective neoepitope exposed only after a specific caspase cleavage within cytokeratin 18 during early apoptosis [32]. Epithelial forms of positivity for this marker include cytoplasmic filamentous staining or granular aggregates as shown in Figure 1A. Viable and necrotic cells and late apoptotic epithelial cells are negative for M30.

The expression of Ki67 as a proliferation marker [33] was first evaluated using the MDA-MB231 breast cancer cell line. Proliferative cells were detected using the specific anti-Ki67 mouse antibody ab8191 (Abcam, Cambridge, UK). For the Ki67 reactivity, the positive nuclear staining was evaluated (Figure 1B).

CK positivity in cytospins of SKBR3 or MDA-MB231 cells was detected using the pancytokeratin rabbit antibody sc-15367 (Santa Cruz Biotechnology, Dallas, TX, USA) [34]–[36]. Cell cytospins were evaluated using the automated image analysis (ARIOL) system CTCs software (Genetix, New Milton, UK) [37].

### Triple immunofluorescence for simultaneous detection of Ki67 and M30

CK-positive and M30-positive or Ki67-positive CTCs were identified by triple immunofluorescence. Briefly, PBMC cytospins were fixed using 100% ice-cold pure methanol (-20°C) for 7 minutes at room temperature (RT). Cell permeabilization was performed with 100% ice-cold pure acetone (-20°C) for 3 minutes and followed by incubation with blocking buffer (PBS/2% FBS) for 30 minutes. Cytospins were washed with PBS and stained with anti-Ki67 mouse antibody diluted 1:50, overnight. This was followed by the secondary anti-mouse Alexa 633 antibody (Molecular Probes, Invitrogen, Carlsbad, CA, USA). Subsequently, cells were stained with the pancytokeratin rabbit antibody sc-15367 (Santa Cruz Biotechnology) diluted 1:50 [34]–[36], followed by the secondary anti-rabbit Alexa 555 (Molecular Probes, Invitrogen). Afterward, cells were stained with M30 fluorescein-conjugated mouse antibody diluted 1:100 for 90 minutes, in order to avoid cross-reaction with the secondary anti-mouse Alexa 633 antibody. Finally, 4’,6-diamidino-2-phenylindole (DAPI) antifade reagent (Invitrogen) was added to each sample for nuclear staining. To ensure the performance of Ki67 and M30 staining, cytospins of MDA-MB231 and SKBR3 staurosporine-treated cells were included in each separate experiment as positive controls. Negative controls, prepared by omitting the corresponding primary antibody and adding the secondary immunoglobulin G (IgG) isotype antibody, were also used in each separate experiment.

**Figure 2:**
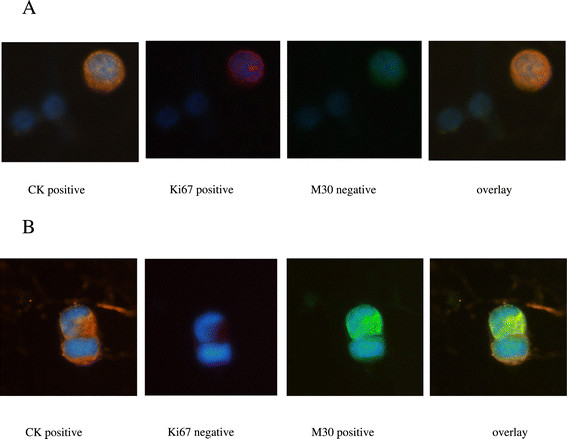
**Expression of the proliferation marker Ki67 and the apoptotic marker M30 in CTCs of patients with early breast cancer. (A)** Representative image of a CTC stained positive for the proliferation marker Ki67 along with PBMCs. **(B)** Representative image of a CTC stained positive for the apoptotic marker M30. Cytospins were triple stained with pancytokeratin rabbit antibody/secondary anti-rabbit Alexa Fluor 555 (orange), anti-Ki67 mouse antibody/secondary anti-mouse Alexa 633 (red) and M30 mouse FITC-conjugated antibody (green). Cell nuclei were stained with DAPI (blue). Images were taken by ARIOL system (X60). ARIOL system, automated image analysis system; CTCs, circulating tumor cells; DAPI, 4’,6-diamidino-2-phenylindole; FITC, fluorescein isothiocyanate; PBMCs, peripheral blood mononuclear cells.

Specific staining was easily distinguished by the differential intracellular distribution of the examined molecules (Figure 2A, B). Moreover, the cytomorphological and immunophenotypic criteria proposed by Meng and colleagues (that is high nuclear to cytoplasmic ratio, cells larger than white blood cells, and so on; [28]) were used to characterize a CK-positive cell as a CTC. In the evaluation of the samples prepared from healthy female donors, CK-negative cells expressing either Ki67 or M30 were detected. On the contrary, there were no CK-positive cells identified in any of these samples. To further confirm the specificity of the method for cytokeratin detection, 10^6^ PBMCs from each CTC-positive patient were subsequently tested with a rabbit control antibody to evaluate nonspecific staining. We were not able to detect any event in these samples fulfilling all the criteria described above for a CTC. All cytospins were evaluated using the ARIOL microscopy system.

### Statistical analysis

Since this is a noninterventional study, there is not a specific statistical estimation of the sample size. Enrolled patients represent a cohort of patients followed in our institution. Data were analyzed using the GraphPad prism software (version 6) (GraphPad Software, San Diego, CA, USA). Frequencies were compared by Fisher’s exact test or chi-square and nonparametric Mann-Whitney test.

## Results

### Detection of CTCs in blood samples of dormancy candidate patients

A cohort of 122 DC patients was evaluated for the presence of CTCs. The median interval from surgical removal of the primary tumor till CTC evaluation was 6 years (range, 5 to 19). CTCs were detected in 40 (33%) out of 122 patients. The demographics of CTC-positive and CTC-negative patients are presented in Table 1.

**Table 1:** **Characteristics of dormancy candidates**

	CTC-positive	CTC-negative	
	(n = 40)	(n = 82)	***P***
**Age, years**			
Median (range)	52 (27-75)	57 (31-71)	0.133
	**N (%)**		
**Menopausal status**			
Premenopausal	21 (52.5)	28 (34.1)	0.152
Postmenopausal	18 (45)	51 (62.2)	
UN	1 (2.5)	3 (3.6)	
**HR status**			
ER(+) and/or PR(+)	26 (65)	48 (58.5)	0.212
ER(-)/PR(-)	12 (30)	24 (29.3)	
UN	2 (5)	10 (12.2)	
**HER2 status**			
HER2(+)	9 (22.5)	12 (14.6)	0.252
HER2(-)	29 (72.5)	59 (72)	
UN	2 (5)	11 (13.4)	
**Tumor size**			
T1	10 (25)	25 (30.5)	0.418
T2	21 (52.5)	45 (54.9)	
T3	8 (20)	8 (9.7)	
UN	1 (2.5)	4 (4.8)	
**Grade**			
I	0 (0)	1 (1.2)	0.938
II	18 (45)	40 (48.8)	
III	17 (42.5)	33 (40.2)	
Lobular	3 (7.5)	5 (6)	
UN	2 (5)	3 (3.6)	
**Number of positive nodes**			
0	15 (37.5)	33 (40.2)	0.220
1-3	12 (30)	33 (40.2)	
≥4	12 (30)	12 (14.6)	
UN	1 (2.5)	4 (4.8)	

CTCs, circulating tumor cells; ER, estrogen receptor; HER2, human epidermal growth factor 2; HR, hormone receptor; PR, progesterone receptor; UN, unknown.

### Expression of M30 and Ki67 on CTCs of dormancy candidate patients

M30, as a marker of apoptosis, and Ki67, as a marker of proliferation, were used to characterize CTCs. In 25 (62.5%) out of 40 CTC-positive patients, all detected CTCs were negative for both Ki67 and M30 (Ki67(-)/M30(-) CTCs) corresponding to dormant cells [5],[30]. In the remaining CTC-positive patients both dormant and nondormant (proliferative or apoptotic) CTCs were identified; seven (17.5%) had proliferative (Ki67(+)/M30(-)) CTCs, four (10%) had apoptotic (Ki67(-)/M30(+)) and four (10%) had both phenotypes besides the dormant sub-population (Table 2).

**Table 2:** **Incidence of proliferative and apoptotic CTCs in CTC-positive DC and metastatic patients and their percentages among the total CTCs detected**

	CTC phenotype (n%)			
**Patient groups**	**I**	**II**	**III**	**IV**
	**Dormant only** ^**a**^	**Proliferative** ^**b**^	**Apoptotic** ^**c**^	**All phenotypes** ^**e**^
**Dormancy candidates (n = 40)**	25 (62.5)	7 (17.5)	4 (10)	4 (10)
**Metastatic patients (n = 15)**	8 (53.4)	7 (46.6)^*^	0 (0)	0 (0)
	**CTC phenotype (n %)**			
	**Dormant** ^**a**^	**Nondormant** ^**d**^		
**Number of CTCs**		**Proliferative** ^**b**^	**Apoptotic** ^**c**^	
**Dormancy candidates (n = 244)**	201 (82.4)	14 (5.7)	29 (11.9)	
**Metastatic patients (n = 142)**	84 (59.1)^**^	58 (40.9)^**^	0 (0)	

Patients with ^a^Ki67(-)/M30(-) CTCs, ^b^Ki67(+)/M30(-) CTCs, ^c^Ki67(-)/M30(+) CTCs, ^d^Ki67(+)/M30(-) or Ki67(-)/M30(+) CTCs and ^e^patients harboring all phenotypes. ^*^P = 0.0394, ^**^P <0.0001, compared to dormancy candidates. CTCs, circulating tumor cells; DC, dormancy candidates.

A total of 244 CTCs were detected in the whole group of DC (mean: 6.1 CTCs/patient, standard error of the mean (SEM) ± 1.8). As shown in Table 2, 82.4% were dormant, 11.9% apoptotic and 5.7% proliferative. Among the nondormant population, the proportions of proliferative/nondormant and apoptotic/nondormant CTCs were 32.5% and 67.5%, respectively. There were no CTCs that could be stained positive for both Ki67 and M30.

Three (12%) out of twenty-five patients (Group I, Table 2) harboring exclusively dormant CTCs, and five (45.4%) out of eleven patients with proliferative CTCs (Groups II and IV, Table 2) experienced late disease relapse (*P* = 0.04); recurrence was detected at 6 to 15 years (mean, 10 years) after surgical removal of the primary tumor.

### Characterization of the proliferative and apoptotic status of CTCs in metastatic breast cancer patients relapsing at least 5 years after surgery

A group of 40 metastatic breast cancer patients who had relapsed after a median of 5 years (range, 5 to 10 years) from surgery were evaluated for the presence of CTCs, prior to the initiation of any systemic first-line therapy. CTCs were detected in 15 (37.5%) patients and dormant CTCs were evident in all but one patient. Eight (53.4%) patients harbored exclusively dormant CTCs, whereas in 46.6% Ki67(+) CTCs were also detected (*P* = 0.039 compared to DC) (Table 2). None of the patients harbored apoptotic CTCs.

A total of 142 CTCs were identified in the whole group (mean: 9.4 CTCs/patient, SEM ± 6.6); 59.1% of these cells were dormant and 40.9% were Ki67(+) (*P* <0.0001 compared to DC) (Table 2). Since no apoptotic CTCs were detected, the proportion of proliferative CTCs among the nondormant population was 100%.

### Incidence of proliferative and apoptotic CTCs in sequential follow-up samples of dormancy candidates

To monitor the kinetics of proliferative and apoptotic CTCs during the period of dormancy, sequential follow-up samples were evaluated in the group of eight out of forty CTC-positive DC who subsequently experienced late disease relapse and in another group of eight DC who remained in a prolonged disease-free status during the whole follow-up period. The last group was selected according to the length of follow-up time and/or the availability of comparable numbers of sequential samples for evaluation. Median disease-free interval from the surgical removal of the primary tumor was 10.5 years (range 6 to 15 years) for the first group, whereas the median follow-up time was 11 years (range 8 to 13 years) for the second group.

i. **Group of DC with late relapse**

A total of 27 serial samples (median three/patient (range two to six)) were evaluated (Additional file 1). Two (25%) of eight patients (#7, #8) had exclusively dormant CTCs during the whole follow-up period, two (25%) (#1, #6) had proliferative CTCs and four (50%) (#2, #3, #4, #5) had proliferative as well as apoptotic CTCs besides the dormant population (Table 3). Among the total CTCs identified in all follow-up samples, 88% were dormant, 6.8% were proliferative and 5.2% apoptotic (Additional file 1). The proportions of proliferative/nondormant and apoptotic/nondormant CTCs were 56.5% and 43.5%, respectively.

**Table 3:** N**umbers of total, proliferative and apoptotic CTCs in serial samples during the dormancy period for DC with late relapse (n = 8)**

Patient number	Dormancy period (yrs)	Test no/(time since surgery yrs)	Status	Total CTCs	Dormant ^a^/Total (%)	Nondormant ^d^/Total (%)	Proliferative ^b^/Nondormant ^d^(%)	Apoptotic ^c^/Nondormant ^d^(%)
**1**	6	1/3 (5y)	DF	1	100	0	0	0
		2/3 (5.5y)	DF	4	87.5	12.5	100	0
		3/3 (6y)	R	3	83.3	16.7	100	0
**2**	7	1/4 (5y)	DF	12	50	50	100	0
		2/4 (5.5y)	DF	215	93	7	80	20
		3/4 (6.5y)	DF	2	50	50	100	0
		4/4 (7y)	R	1	0	0	100	0
**3**	10.5	1/5 (5.5y)	DF	0	0	0	0	0
		2/5 (6y)	DF	0	0	0	0	0
		3/5 (8y)	DF	22	50	50	36.4	63.6
		4/5 (10y)	DF	0	0	0	0	0
		5/5 (10.5y)	R	0.5	100	0	0	0
**4**	9.5	1/5 (5y)	DF	1	100	0	0	0
		2/5 (5.5y)	DF	9	89	11	0	100
		3/5 (6.5y)	DF	57	91.3	8.7	20	80
		4/5 (7.5y)	DF	1	100	0	0	0
		5/5 (9.5y)	R	0	0	0	0	0
**5**	11	1/4 (5y)	DF	9.5	100	0	0	0
		2/4 (6y)	DF	38	87.3	12.7	3.5	96.5
		3/4 (10y)	DF	0	0	0	0	0
		4/4 (11y)	R	0	0	0	0	0
**6**	10.5	1/7 (5.5y)	DF	0	0	0	0	0
		2/7 (7y)	DF	0	0	0	0	0
		3/7 (7.5y)	DF	0	0	0	0	0
		4/7 (8y)	DF	0	0	0	0	0
		5/7 (9y)	DF	2.5	60	40	100	0
		6/7 (10y)	DF	0.5	100	0	0	0
		7/7 (10.5y)	R	9	50	50	100	0
**7**	15	1/3 (11y)	DF	5	100	0	0	0
		2/3 (12y)	DF	2	100	0	0	0
		3/3 (15y)	R	0.5	100	0	0	0
**8**	11	1/4 (6.5y)	DF	0	0	0	0	0
		2/4 (7y)	DF	0	0	0	0	0
		3/4 (7.5y)	DF	1	100	0	0	0
		4/4 (11y)	R	0.5	100	0	0	0

^a^Ki67(-)/M30(-) CTCs, ^b^Ki67(+)/M30(-) CTCs, ^c^Ki67(-)/M30(+), ^d^Ki67(+)/M30(-) or Ki67(-)/M30(+) CTCs. CTCs, circulating tumor cells; DC, dormancy candidates; DF, disease-free; R, on relapse.

ii. **Group of DC with prolonged disease-free status**

A total of 36 sequential samples were analyzed (Additional file 1). Four (50%) of eight DC (#9, #13, #14, #15) had exclusively dormant CTCs during the whole follow-up period, one (12.5%) (#10) had proliferative, one (12.5%) (#16) had apoptotic and two (25%) (#11, #12) had both populations CTCs beside the dormant one (Table 4). Α total of 77 CTCs were detected in all samples. Among them, 78% were dormant (*P* = 0.028 compared to the relapsed group), 6.5% were proliferative and 15.8% apoptotic (*P* = 0.0029 compared to the relapsed group) (Additional file 1). The proportions of proliferative/nondormant and apoptotic/nondormant CTCs were 29.4% and 70.6%, respectively (*P* = 0.0002 compared to the relapsed group).

**Table 4:** **Numbers of total, proliferative and apoptotic CTCs in serial samples during the dormancy period for DC with a prolonged disease-free status (n = 8)**

Patient number	Dormancy period (yrs)	Test no/(time since surgery yrs)	Status	Total CTCs	Dormant ^a^/Total	Nondormant ^d^/Total (%)	Proliferative ^b^/Nondormant ^d^(%)	Apoptotic ^c^/Nondormant ^d^(%)
**9**	8	1 (5)	DF	1	100	0	0	0
		2 (5.5)	DF	0	0	0	0	0
		3 (6)	DF	1	100	0	0	0
		4 (6.5)	DF	0	0	0	0	0
		5 (7)	DF	0	0	0	0	0
		6 (7.5)	DF	0	0	0	0	0
		7 (8)	DF	0	0	0	0	0
**10**	13	1 (5)	DF	1.5	100	0	0	0
		2 (8)	DF	0	0	0	0	0
		3 (11)	DF	5	60	40	100	0
		4 (12)	DF	0.5	100	0	0	0
		5 (13)	DF	0.5	0	100	100	0
**11**	10	1 (7)	DF	1	0	100	0	100
		2 (8)	DF	1	0	100	0	100
		3 (9)	DF	2	50	50	100	0
		4 (10)	DF	2	100	0	0	0
**12**	13	1 (5)	DF	2	75	25	100	0
		2 (5.5)	DF	3.5	100	0	0	0
		3 (9.5)	DF	22	75	25	25	75
		4 (10.5)	DF	1	0	100	0	100
		5 (11.5)	DF	0	0	0	0	0
		6 (12)	DF	0	0	0	0	0
		7 (13)	DF	0	0	0	0	0
**13**	10.5	1 (5)	DF	0.5	100	0	0	0
		2 (9)	DF	1	100	0	0	0
		3 (10.5)	DF	0	0	0	0	0
**14**	10.5	1 (5)	DF	0	0	0	0	0
		2 (6)	DF	0.5	100	0	0	0
		3 (10)	DF	0	0	0	0	0
**15**	8	1 (5)	DF	0.5	100	0	0	0
		2 (5.5)	DF	0	0	0	0	0
		3 (6)	DF	0.5	100	0	0	0
		4 (8)	DF	0	0	0	0	0
**16**	12	1 (10)	DF	0	0	0	0	0
		2 (11)	DF	1	100	0	0	0
		3 (12)	DF	31	82	18	0	100

^a^Ki67(-)/M30(-) CTCs, ^b^Ki67(+)/M30(-) CTCs, ^c^Ki67(-)/M30(+), ^d^Ki67(+)/M30(-) or Ki67(-)/M30(+) CTCs. CTCs, circulating tumor cells; DC, dormancy candidates; DF, disease-free.

### Evaluation of Ki67 and M30 status of CTCs at the time points with significantly increased CTC numbers during follow-up

As shown in Tables 3 and 4, a significant increase in CTC counts was observed on six of the thirty-eight CTC-positive follow-up samples. The mean CTC number at the time of highest CTC value was 64 (SEM ± 30.6) compared to 2.4 (SEM ± 0.5) for the remaining CTC positive samples (*P* = 0.0001).

This robust increase in CTC counts, ranging from 22 to 215 CTCs, was observed in four (50%) out of eight DC with subsequent late relapse (#2, #3, #4, #5, Table 3). Patient #2 with 80% of the nondormant CTC population expressing Ki67 at the time of the highest CTC count relapsed 1.5 years later. On the contrary, in the other three patients (#3, #4 and #5), presenting higher percentages of apoptotic (63.6%, 80%, 96.5%) compared to proliferative nondormant CTCs, relapses occurred 2.5, 3 and 5 years later (Additional file 2A).

In the group of patients that remained in prolonged disease-free status during the follow-up period, only two (25%) (#12 and #16, Additional file 2B) presented a significant increase in CTC counts (22 and 31 CTCs, respectively) at 3.5 and 0 years prior to the last evaluation; in these patients 75% and 100% of the nondormant CTC population was apoptotic. The remaining patients presented low CTC counts of dormant cells.

## Discussion

In the current study we observed that many patients with breast cancer continue to have CTCs beyond 5 years after surgery, despite the absence of clinical evidence of disease recurrence. Herein we provide, to our knowledge for the first time, an insight into the proliferative and apoptotic status of these CTCs; we show that their great majority are neither proliferative nor apoptotic, possibly representing dormant cells. In addition, we demonstrate that these patients also harbor nondormant CTCs and that the dormancy state might be linked to increased apoptosis, whereas escape from dormancy is associated with increased proliferative index in CTCs.

In this trial, we focused in the detection and characterization of CTCs in early breast cancer patients beyond the time frame of the first 5 years of follow up. These patients were characterized as dormancy candidates since they are likely to have CTCs in a dormant state and, at the same time, are still at considerable risk for recurrence and death from their disease. We used a triple immunofluorescence technique in order to detect CK-positive cells on PBMC cytospins and to evaluate their proliferative and apoptotic status after staining with Ki67 and M30 antibodies, respectively [38]. CTCs were detected in 33% of 122 DC, in line with the report by Meng *et al*. [28] where 36% of 36 dormancy candidates had CTCs detected by immunocytochemistry, 7 to 22 years following surgery. Similarly, Payne *et al.* [39] reported that measures of minimal residual disease, including CTCs, were evident in patients with primary breast cancer more than 4 years following surgical treatment.

Another objective of this study was the characterization of CTCs in DC according to their proliferative and apoptotic status. It is generally accepted that the majority of disseminated tumor cells found in the bone marrow or the circulation of breast cancer patients are nonproliferative cells [40],[41]. In addition, different rates of apoptosis in DTCs or CTCs have been reported according to the tumor type, the disease stage and/or phase of treatment [42]–[46]. In our study, CTCs lacking staining for the markers Ki67 or M30, which are used to identify proliferative and apoptotic CTCs, respectively, were characterized as dormant [5],[30]. It should be mentioned here that, although it is generally accepted that Ki67 protein expression and cell proliferation are closely linked, the dynamic expression of Ki67 in a cell determined as Ki67 negative, cannot be ruled out. Indeed, it has been suggested that Ki67 may remain undetectable during the G1 phase, therefore, the cell under evaluation could be incorrectly characterized as nonproliferative [47].

We showed that 27.5% and 20% of DC patients harbor proliferative and apoptotic CTCs, respectively. However, most CTCs identified in the whole group were dormant, whereas among the nondormant population, apoptotic CTCs prevailed. Since the half-life of CTCs has been estimated between 1 and 2.4 hours [28], our observations are in line with the hypothesis that, during dormancy, CTCs are most probably derived from undetected micrometastatic deposits where a balance between proliferation and apoptosis, in favor of the latter, exists [4],[7]. Similarly, in the report by Payne *et al*., apoptosis, as demonstrated by an increase in larger-sized fragments in cell-free DNA, was inversely related to the detection of DTCs in the bone marrow, suggesting that this measure of micrometastatic disease probably emerged from dying micrometastases [39]. In preclinical models, apoptosis, through impaired vascularization or immune-mediated mechanisms, has been considered to be involved in the regulation of dormancy [4],[7],[26]. Interestingly, in a mouse model of primary chemical carcinogenesis, the immune system was shown to restrain the net expansion of ‘dormant’ tumor cells and this was characterized by a combination of increased apoptosis and decreased tumor cell proliferation [48]. However, the simultaneous presence of DTCs prone to mechanisms of cellular dormancy cannot be excluded.

In our study, metastatic patients who recurred after 5.5 to 10 years following surgery, were included as a control group for the evaluation of CTCs in a condition that resembles escape from dormancy. Although a relatively low CTC detection rate was observed among those patients [49], possibly related to the limited number of patients analyzed, it was shown that none had apoptotic CTCs at the time of disease relapse. This is in agreement with the study by Fehm *et al*., where no apoptotic DTCs were detected in patients with tumor progression after neoadjuvant chemotherapy [42]. In addition, in metastatic patients, dormant CTC counts were decreased and the proliferative index in CTCs was increased as compared to dormancy candidates. Moreover, it is also of interest that subsequent late relapses were recorded in 45.4% out of DC patients with proliferative CTCs compared to only 12% of DC harboring exclusively dormant CTCs. Thus, escape from dormancy and late relapse could be associated with increased proliferation in CTCs. In accordance, studies using preclinical models suggest that escape from dormancy is associated with cellular proliferation driven by microenvironmental signals leading to extracellular signal-related kinase (ERK) activation [50],[51].

We further evaluated the kinetics and the phenotype of CTCs in sequential samples obtained during follow-up from two groups of DC; from one that presented late relapse during follow-up and one that remained in a prolonged disease-free status. This was considered of importance since the fluctuations of these markers over time had not been previously described, and could be informative regarding subsequent disease recurrence.

It was shown that CTCs were intermittently present during the evaluation period albeit at similar frequency among patients in both groups. Interestingly, the group of DC with prolonged disease-free status generally presented lower CTC counts on serial samples compared to the group with late relapse, and this could be related to the maintenance of dormancy. On the other hand, it seems that the repetitive detection of CTCs does not necessarily indicate future relapse and points to the need for further characterization of these CTCs. Thus, the apoptotic index prevailed in the group with prolonged disease-free status (that maintained dormancy). Another interesting finding was that several patients presented, at some point during follow-up, a robust increase in CTC numbers involving both dormant and nondormant CTCs. The balance between proliferation and apoptosis at this time point could be associated with the time to subsequent disease recurrence.

Our study has several limitations that should be considered. First, an overlap was observed in the detection of proliferation and apoptosis markers between the relapse-free DC and those who relapsed, representing a significant limitation for their clinical use, especially when a sample from a single time point is evaluated. Another limitation is the retrospective nature of our study, the small number of patients followed with serial samples, and the lack of matching in terms of patient and disease characteristics between the two groups. Moreover, it could be argued that the actual CTC phenotype could not be reliably depicted in cases with low CTC counts. Nevertheless, this is the real case scenario, since in general, low CTC counts are observed in patients with early disease [14]. Finally, although in breast cancer the immunohistochemical assessment of proliferation using the marker Ki67 is considered important, both in clinical practice and research, substantial interlaboratory variability limits its clinical use [52]. It is conceivable that Ki67 evaluation of isolated cells could be prone to similar drawbacks.

## Conclusions

Our data suggest that breast cancer dormancy displays significant differences compared to the overt metastatic state regarding the incidence of dormant and nondormant CTCs as well as the balance between proliferation and apoptosis in CTCs. Moreover, in each patient, the dormancy period seems to be characterized by variations in CTC load, in the shift between dormant and nondormant populations and in the balance between proliferative and apoptotic nondormant CTCs. This balance could be associated with the maintenance of or the escape from clinical dormancy. However, the observed variability in CTC detection rate as well as in the expression of these parameters over time, even among the same individual, precludes firm conclusions to be drawn regarding their use in the prediction of patient prognosis, at least as they stand in the current study. Our findings are rather hypothesis generating and merit further investigation in larger studies designed to evaluate their clinical significance, either alone or as part of a prognostic model, in order to define high-risk patient subgroups that might benefit from extended or secondary adjuvant treatments.

## Additional files

## Electronic supplementary material

Additional file 1: Serial sample evaluation in dormancy candidates. (DOC 36 KB)

Additional file 2: **Ki67(+) and M30(+) CTCs numbers in the follow up samples with significantly increased CTC numbers in relapsed** **(A)** **and relapse-free** **(B)** **dormancy candidates.** (DOC 40 KB)

Below are the links to the authors’ original submitted files for images.

Authors’ original file for figure 1

Authors’ original file for figure 2

## Abbreviations

ARIOL system: automated image analysis system
CK: cytokeratin
CTCs: circulating tumor cells
DAPI: 4’,6-diamidino-2-phenylindole
DC: dormancy candidates
DMEM: Dulbecco’s modified Eagle’s medium
DTCs: disseminated tumor cells
FBS: fetal bovine serum
FITC: fluorescein isothiocyanate
PBMCs: peripheral blood mononuclear cells
PBS: phosphate-buffered saline
SEM: standard error of the mean

## References

[1] Karrison TG, Ferguson DJ, Meier P (1999). Dormancy of mammary carcinoma after mastectomy. J Natl Cancer Inst.

[2] Rosen PP, Groshen S, Kinne DW (1991). Prognosis in T2N0M0 stage I breast carcinoma: a 20-year follow-up study. J Clin Oncol.

[3] Saphner T, Tormey DC, Gray R (1996). Annual hazard rates of recurrence for breast cancer after primary therapy. J Clin Oncol.

[4] Aguirre-Ghiso JA (2007). Models, mechanisms and clinical evidence for cancer dormancy. Nat Rev Cancer.

[5] Luzzi KJ, MacDonald IC, Schmidt EE, Kerkvliet N, Morris VL, Chambers AF, Groom AC (1998). Multistep nature of metastatic inefficiency: dormancy of solitary cells after successful extravasation and limited survival of early micrometastases. Am J Pathol.

[6] Naumov GN, MacDonald IC, Weinmeister PM, Kerkvliet N, Nadkarni KV, Wilson SM, Morris VL, Groom AC, Chambers AF (2002). Persistence of solitary mammary carcinoma cells in a secondary site: a possible contributor to dormancy. Cancer Res.

[7] Holmgren L, O’Reilly MS, Folkman J (1995). Dormancy of micrometastases: balanced proliferation and apoptosis in the presence of angiogenesis suppression. Nat Med.

[8] Demicheli R, Terenziani M, Valagussa P, Moliterni A, Zambetti M, Bonadonna G (1994). Local recurrences following mastectomy: support for the concept of tumor dormancy. J Natl Cancer Inst.

[9] Demicheli R, Abbattista A, Miceli R, Valagussa P, Bonadonna G (1996). Time distribution of the recurrence risk for breast cancer patients undergoing mastectomy: further support about the concept of tumor dormancy. Breast Cancer Res Treat.

[10] Hanin LG, Zaider M (2010). Cell-survival probability at large doses: an alternative to the linear-quadratic model. Phys Med Biol.

[11] Braun S, Vogl FD, Naume B, Janni W, Osborne MP, Coombes RC, Schlimok G, Diel IJ, Gerber B, Gebauer G, Pierga JY, Marth C, Oruzio D, Wiedswang G, Solomayer EF, Kundt G, Strobl B, Fehm T, Wong GY, Bliss J, Vincent-Salomon A, Pantel K (2005). A pooled analysis of bone marrow micrometastasis in breast cancer. N Engl J Med.

[12] Daskalaki A, Agelaki S, Perraki M, Apostolaki S, Xenidis N, Stathopoulos E, Kontopodis E, Hatzidaki D, Mavroudis D, Georgoulias V (2009). Detection of cytokeratin-19 mRNA-positive cells in the peripheral blood and bone marrow of patients with operable breast cancer. Br J Cancer.

[13] Ignatiadis M, Xenidis N, Perraki M, Apostolaki S, Politaki E, Kafousi M, Stathopoulos EN, Stathopoulou A, Lianidou E, Chlouverakis G, Sotiriou C, Georgoulias V, Mavroudis D (2007). Different prognostic value of cytokeratin-19 mRNA positive circulating tumor cells according to estrogen receptor and HER2 status in early-stage breast cancer. J Clin Oncol.

[14] Rack B, Schindlbeck C, Juckstock J, Andergassen U, Hepp P, Zwingers T, Friedl TW, Lorenz R, Tesch H, Fasching PA, Fehm T, Schneeweiss A, Lichtenegger W, Beckmann MW, Friese K, Pantel K, Janni W (2014). Circulating tumor cells predict survival in early average-to-high risk breast cancer patients. J Natl Cancer Inst.

[15] Stathopoulou A, Vlachonikolis I, Mavroudis D, Perraki M, Kouroussis C, Apostolaki S, Malamos N, Kakolyris S, Kotsakis A, Xenidis N, Reppa D, Georgoulias V (2002). Molecular detection of cytokeratin-19-positive cells in the peripheral blood of patients with operable breast cancer: evaluation of their prognostic significance. J Clin Oncol.

[16] Xenidis N, Perraki M, Kafousi M, Apostolaki S, Bolonaki I, Stathopoulou A, Kalbakis K, Androulakis N, Kouroussis C, Pallis T, Christophylakis C, Argyraki K, Lianidou ES, Stathopoulos S, Georgoulias V, Mavroudis D (2006). Predictive and prognostic value of peripheral blood cytokeratin-19 mRNA-positive cells detected by real-time polymerase chain reaction in node-negative breast cancer patients. J Clin Oncol.

[17] Janni W, Rack B, Schindlbeck C, Strobl B, Rjosk D, Braun S, Sommer H, Pantel K, Gerber B, Friese K (2005). The persistence of isolated tumor cells in bone marrow from patients with breast carcinoma predicts an increased risk for recurrence. Cancer.

[18] Janni W, Vogl FD, Wiedswang G, Synnestvedt M, Fehm T, Juckstock J, Borgen E, Rack B, Braun S, Sommer H, Solomayer E, Pantel K, Nesland J, Friese K, Naume B (2011). Persistence of disseminated tumor cells in the bone marrow of breast cancer patients predicts increased risk for relapse–a European pooled analysis. Clin Cancer Res.

[19] Quintela-Fandino M, Lopez JM, Hitt R, Gamarra S, Jimeno A, Ayala R, Hornedo J, Guzman C, Gilsanz F, Cortes-Funes H (2006). Breast cancer-specific mRNA transcripts presence in peripheral blood after adjuvant chemotherapy predicts poor survival among high-risk breast cancer patients treated with high-dose chemotherapy with peripheral blood stem cell support. J Clin Oncol.

[20] Slade MJ, Payne R, Riethdorf S, Ward B, Zaidi SA, Stebbing J, Palmieri C, Sinnett HD, Kulinskaya E, Pitfield T, McCormack RT, Pantel K, Coombes RC (2009). Comparison of bone marrow, disseminated tumour cells and blood-circulating tumour cells in breast cancer patients after primary treatment. Br J Cancer.

[21] Wiedswang G, Borgen E, Karesen R, Qvist H, Janbu J, Kvalheim G, Nesland JM, Naume B (2004). Isolated tumor cells in bone marrow three years after diagnosis in disease-free breast cancer patients predict unfavorable clinical outcome. Clin Cancer Res.

[22] Xenidis N, Ignatiadis M, Apostolaki S, Perraki M, Kalbakis K, Agelaki S, Stathopoulos EN, Chlouverakis G, Lianidou E, Kakolyris S, Georgoulias V, Mavroudis D (2009). Cytokeratin-19 mRNA-positive circulating tumor cells after adjuvant chemotherapy in patients with early breast cancer. J Clin Oncol.

[23] Xenidis N, Perraki M, Apostolaki S, Agelaki S, Kalbakis K, Vardakis N, Kalykaki A, Xyrafas A, Kakolyris S, Mavroudis D, Georgoulias V (2013). Differential effect of adjuvant taxane-based and taxane-free chemotherapy regimens on the CK-19 mRNA-positive circulating tumour cells in patients with early breast cancer. Br J Cancer.

[24] Jatoi I, Tsimelzon A, Weiss H, Clark GM, Hilsenbeck SG (2005). Hazard rates of recurrence following diagnosis of primary breast cancer. Breast Cancer Res Treat.

[25] Early Breast Cancer Trialists’ Collaborative Group (EBCTCG) (2005). Effects of chemotherapy and hormonal therapy for early breast cancer on recurrence and 15-year survival: an overview of the randomised trials. Lancet.

[26] Uhr JW, Pantel K (2011). Controversies in clinical cancer dormancy. Proc Natl Acad Sci U S A.

[27] van der Sangen MJ, van de Wiel FM, Poortmans PM, Tjan-Heijnen VC, Nieuwenhuijzen GA, Roumen RM, Ernst MF, Tutein Nolthenius-Puylaert MC, Voogd AC (2011). Are breast conservation and mastectomy equally effective in the treatment of young women with early breast cancer? Long-term results of a population-based cohort of 1,451 patients aged </= 40 years. Breast Cancer Res Treat.

[28] Meng S, Tripathy D, Frenkel EP, Shete S, Naftalis EZ, Huth JF, Beitsch PD, Leitch M, Hoover S, Euhus D, Haley B, Morrison L, Fleming TP, Herlyn D, Terstappen LW, Fehm T, Tucker TF, Lane N, Wang J, Uhr JW (2004). Circulating tumor cells in patients with breast cancer dormancy. Clin Cancer Res.

[29] Saloustros E, Perraki M, Apostolaki S, Kallergi G, Xyrafas A, Kalbakis K, Agelaki S, Kalykaki A, Georgoulias V, Mavroudis D (2011). Cytokeratin-19 mRNA-positive circulating tumor cells during follow-up of patients with operable breast cancer: prognostic relevance for late relapse. Breast Cancer Res.

[30] Cameron MD, Schmidt EE, Kerkvliet N, Nadkarni KV, Morris VL, Groom AC, Chambers AF, MacDonald IC (2000). Temporal progression of metastasis in lung: cell survival, dormancy, and location dependence of metastatic inefficiency. Cancer Res.

[31] Tang D, Lahti JM, Kidd VJ (2000). Caspase-8 activation and bid cleavage contribute to MCF7 cellular execution in a caspase-3-dependent manner during staurosporine-mediated apoptosis. J Biol Chem.

[32] Ueno T, Toi M, Linder S (2005). Detection of epithelial cell death in the body by cytokeratin 18 measurement. Biomed Pharmacother.

[33] Gerdes J, Schwab U, Lemke H, Stein H (1983). Production of a mouse monoclonal antibody reactive with a human nuclear antigen associated with cell proliferation. Int J Cancer.

[34] Ignatiadis M, Kallergi G, Ntoulia M, Perraki M, Apostolaki S, Kafousi M, Chlouverakis G, Stathopoulos E, Lianidou E, Georgoulias V, Mavroudis D (2008). Prognostic value of the molecular detection of circulating tumor cells using a multimarker reverse transcription-PCR assay for cytokeratin 19, mammaglobin A, and HER2 in early breast cancer. Clin Cancer Res.

[35] Kallergi G, Mavroudis D, Georgoulias V, Stournaras C (2007). Phosphorylation of FAK, PI-3 K, and impaired actin organization in CK-positive micrometastatic breast cancer cells. Mol Med.

[36] Kallergi G, Agelaki S, Kalykaki A, Stournaras C, Mavroudis D, Georgoulias V (2008). Phosphorylated EGFR and PI3K/Akt signaling kinases are expressed in circulating tumor cells of breast cancer patients. Breast Cancer Res.

[37] Kallergi G, Papadaki MA, Politaki E, Mavroudis D, Georgoulias V, Agelaki S (2011). Epithelial to mesenchymal transition markers expressed in circulating tumour cells of early and metastatic breast cancer patients. Breast Cancer Res.

[38] Kallergi G, Konstantinidis G, Markomanolaki H, Papadaki MA, Mavroudis D, Stournaras C, Georgoulias V, Agelaki S (2013). Apoptotic circulating tumor cells (CTCs) in early and metastatic breast cancer patients. Mol Cancer Ther.

[39] Payne RE, Hava NL, Page K, Blighe K, Ward B, Slade M, Brown J, Guttery DS, Zaidi SA, Stebbing J, Jacob J, Yague E, Shaw JA, Coombes RC (2012). The presence of disseminated tumour cells in the bone marrow is inversely related to circulating free DNA in plasma in breast cancer dormancy. Br J Cancer.

[40] Muller V, Stahmann N, Riethdorf S, Rau T, Zabel T, Goetz A, Janicke F, Pantel K (2005). Circulating tumor cells in breast cancer: correlation to bone marrow micrometastases, heterogeneous response to systemic therapy and low proliferative activity. Clin Cancer Res.

[41] Pantel K, Schlimok G, Braun S, Kutter D, Lindemann F, Schaller G, Funke I, Izbicki JR, Riethmuller G (1993). Differential expression of proliferation-associated molecules in individual micrometastatic carcinoma cells. J Natl Cancer Inst.

[42] Fehm T, Becker S, Becker-Pergola G, Sotlar K, Gebauer G, Durr-Storzer S, Neubauer H, Wallwiener D, Solomayer EF (2006). Presence of apoptotic and nonapoptotic disseminated tumor cells reflects the response to neoadjuvant systemic therapy in breast cancer. Breast Cancer Res.

[43] Hou JM, Krebs MG, Lancashire L, Sloane R, Backen A, Swain RK, Priest LJ, Greystoke A, Zhou C, Morris K, Ward T, Blackhall FH, Dive C (2012). Clinical significance and molecular characteristics of circulating tumor cells and circulating tumor microemboli in patients with small-cell lung cancer. J Clin Oncol.

[44] Larson CJ, Moreno JG, Pienta KJ, Gross S, Repollet M, O’hara SM, Russell T, Terstappen LW (2004). Apoptosis of circulating tumor cells in prostate cancer patients. Cytometry A.

[45] Rossi E, Basso U, Celadin R, Zilio F, Pucciarelli S, Aieta M, Barile C, Sava T, Bonciarelli G, Tumolo S, Ghiotto C, Magro C, Jirillo A, Indraccolo S, Amadori A, Zamarchi R (2010). M30 neoepitope expression in epithelial cancer: quantification of apoptosis in circulating tumor cells by Cell Search analysis. Clin Cancer Res.

[46] Wimberger P, Heubner M, Otterbach F, Fehm T, Kimmig R, Kasimir-Bauer S (2007). Influence of platinum-based chemotherapy on disseminated tumor cells in blood and bone marrow of patients with ovarian cancer. Gynecol Oncol.

[47] Scholzen T, Gerdes J (2000). The Ki-67 protein: from the known and the unknown. J Cell Physiol.

[48] Koebel CM, Vermi W, Swann JB, Zerafa N, Rodig SJ, Old LJ, Indraccolo S, Amadori A, Zamarchi R, Schreiber RD (2007). Adaptive immunity maintains occult cancer in an equilibrium state. Nature.

[49] Cristofanilli M, Budd GT, Ellis MJ, Stopeck A, Matera J, Miller MC, Reuben JM, Doyle GV, Allard WJ, Terstappen LW, Hayes DF (2004). Circulating tumor cells, disease progression, and survival in metastatic breast cancer. N Engl J Med.

[50] Aguirre-Ghiso JA, Liu D, Mignatti A, Kovalski K, Ossowski L (2001). Urokinase receptor and fibronectin regulate the ERK(MAPK) to p38(MAPK) activity ratios that determine carcinoma cell proliferation or dormancy in vivo. Mol Biol Cell.

[51] Aguirre-Ghiso JA, Estrada Y, Liu D, Ossowski L (2003). ERK(MAPK) activity as a determinant of tumor growth and dormancy; regulation by p38(SAPK). Cancer Res.

[52] Polley MY, Leung SC, McShane LM, Gao D, Hugh JC, Mastropasqua MG, Viale G, Zabaglo LA, Penault-Llorca F, Bartlett JM, Gown AM, Symmans WF, Piper T, Mehl E, Enos RA, Hayes DF, Dowsett M, Nielsen TO (2013). An international Ki67 reproducibility study. J Natl Cancer Inst.

