# Supplementary material for: Evaluation of proliferation and apoptosis markers in circulating tumor cells of women with early breast cancer who are candidates for tumor dormancy
Source: Breast Cancer Res. 2014 Nov 29;16:485. doi: 10.1186/s13058-014-0485-8 (PMC4303210; doi:10.1186/s13058-014-0485-8)
Supplement: Supplementary file 1 — Additional file 1: Serial sample evaluation in dormancy candidates. (DOC 36 KB) [file 13058_2014_485_MOESM1_ESM.doc]

**Additional file 1.** Serial sample evaluation in dormancy candidates

|  | **Relapsed group**  **(n=8)** | **Non-relapsed group (n=8)** |  |
| --- | --- | --- | --- |
| **DFI years /FU-time years** median, range | 10.5 (6-15) | 11 (8-13) | *P*=0.159 |
| **Total number of samples** | 27 | 36 |  |
| **CTC positive samples** (n%) | 17 (63) | 21 (58) | *P*=0.797 |
| **CTC-positive sample rate /patient (%)**  Median, range | 83 (25-100) | 62 (25-100) |  |
| **Total CTCs detected**  Median, range | 382  4 (1-215) | 77  1 (0.5-31) |  |
| **Dormant^a^ CTCs (n%)** | 336 (88) | 60 (78) | *P*=0.028 |
| **Non-Dormant^d^ CTCs (n%)**  Proliferative ^b^  Apoptotic^c^ | 46 (12)  26 (6.8)  20 (5.2) | 17 (22)  5 (6.5)  12 (15.5) | *P*=0.0029 |

**^a^**Ki67(-)/M30(-) CTCs, **^b^**Ki67(+)/M30(-) CTCs, **^c^**Ki67(-)/M30(+) CTCs, **^d^**Ki67(+)/M30(-) or Ki67(-)/M30(+) CTCs, DFI= Disease free interval, FU-time = follow-up time.
