# Supplementary material for: Evaluation of proliferation and apoptosis markers in circulating tumor cells of women with early breast cancer who are candidates for tumor dormancy
Source: Breast Cancer Res. 2014 Nov 29;16:485. doi: 10.1186/s13058-014-0485-8 (PMC4303210; doi:10.1186/s13058-014-0485-8)
Supplement: Supplementary file 2 — Additional file 2: Ki67(+) and M30(+) CTCs numbers in the follow up samples with significantly increased CTC numbers in relapsed (A) and relapse-free (B) dormancy candidates. (DOC 40 KB) [file 13058_2014_485_MOESM2_ESM.doc]

**Additional file 2.** Ki67(+) and M30(+) CTCs numbers in the follow up samples with significantly increased CTC

numbers in relapsed **(A)** and relapse-free **(B)** dormancy candidates.

**2**A

|  |  |  |  |  |  |  |  |  |
| --- | --- | --- | --- | --- | --- | --- | --- | --- |
| **Relapsed Patient nu** | **Dormancy Period (yrs)** | **Test no/ time**  **since surgery (yrs)** | **Time to Relapse**  **(yrs)** | **Total**  **CTCs** | **Dormant^a^ /**  **Total (%)** | **Non-Dormant^d^ / Total (%)** | **Proliferative^b^ /**  **Non-Dormant^d^ (%)** | **Apoptotic^c^/**  **Non-Dormant^d^(%)** |
| **2** | 7y | 2/4 (5.5y) | 1.5 | 215 | 93 | 7 | 80 | 20 |
| **3** | 10.5y | 3/5 (8y) | 2.5 | 22 | 50 | 50 | 36.4 | 63.6 |
| **4** | 9.5y | 3/5 (6.5y) | 3 | 57 | 91.3 | 8.7 | 20 | 80 |
| **5** | 11y | 2/4 (6y) | 5 | 38 | 87.3 | 12.7 | 3.4 | 96.5 |

**2**B

| **Non-Relapsed Patient nu** | **Dormancy**  **Period (yrs)** | **Test no/ time**  **since surgery (yrs)** | **Time to last follow-up (yrs)** | **Total CTCs** | **Dormant^a^ /**  **Total (%)** | **Non-Dormant^d^ /**  **Total (%)** | **Proliferative^b^ /**  **Non-Dormant^d^ (%)** | **Apoptotic^c^/**  **Non-Dormant^d^ (%)** |
| --- | --- | --- | --- | --- | --- | --- | --- | --- |
| **12** | 13y | 3 (9.5) | 3.5 | 22 | 75 | 25 | 25 | 75 |
| **16** | 12y | 3 (12) | 0 | 31 | 82 | 18 | 0 | 100 |

**^a^** Ki67(-)/M30(-) CTCs, **^b^** Ki67(+)/M30(-) CTCs, **^c^** Ki67(-)/M30(+), **^d^** Ki67(+)/M30(-) or Ki67(-)/M30(+) CTCs
