# Supplementary figures and images for: Evaluation of proliferation and apoptosis markers in circulating tumor cells of women with early breast cancer who are candidates for tumor dormancy
Source: Breast Cancer Res. 2014 Nov 29;16:485. doi: 10.1186/s13058-014-0485-8 (PMC4303210; doi:10.1186/s13058-014-0485-8)

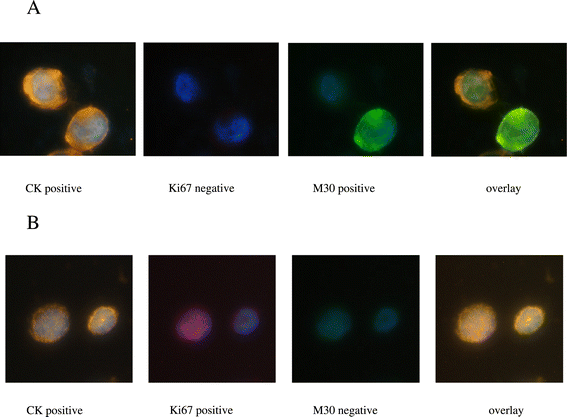

Supplement: Supplementary file 3 — Authors’ original file for figure 1 [file 13058_2014_485_MOESM3_ESM.gif]

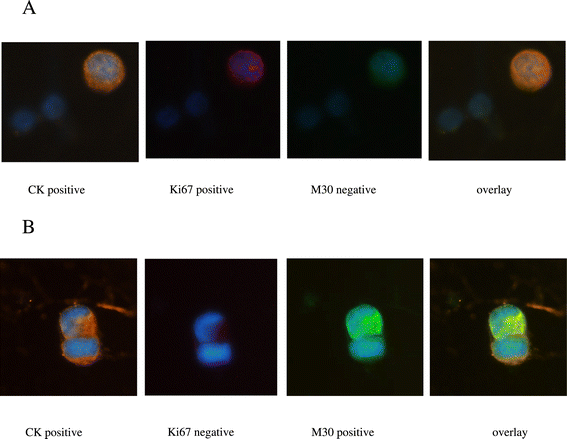

Supplement: Supplementary file 4 — Authors’ original file for figure 2 [file 13058_2014_485_MOESM4_ESM.gif]
